# Using Yoda-1 to mimic laminar flow *in vitro*: A tool to simplify drug testing

**DOI:** 10.1016/j.bcp.2019.08.013

**Published:** 2019-10

**Authors:** Jessica E. Davies, Dora Lopresto, Bonita H.R. Apta, Zhiyuan Lin, Wenxin Ma, Matthew T. Harper

**Affiliations:** Department of Pharmacology, University of Cambridge, Cambridge, UK

**Keywords:** Shear stress, Endothelium, Yoda-1, Toxicology, Laminar flow, Doxorubicin

## Abstract

The endothelium is an attractive drug target and an important site of adverse drug reactions. Endothelial dysfunction is strongly associated with inflammation and contributes to drug-induced cardiovascular toxicity. Endothelial cells in the circulation are exposed to haemodynamic forces including shear stress. Including shear stress may improve future endothelial cell drug discovery or toxicity screening. Piezo-1 is required for endothelial cells to respond to shear stress. In this study, we investigated whether a small molecule activator of Piezo-1, Yoda-1, can mimic the effect of laminar flow-induced shear stress on endothelial cell inflammation, and endothelial cytotoxicity in response to the chemotherapy agent, doxorubicin.

First, we tested whether Yoda-1 could mimic the effects of shear stress of expression of the endothelial adhesion molecules, ICAM-1 and VCAM-1. Human umbilical vein endothelial cells (HUVEC) were cultured in static conditions (with or without Yoda-1) or under laminar flow-induced shear stress (5 dyn/cm^2^). Yoda-1 and laminar flow had similar anti-inflammatory effects, reducing the ability of TNF-α to induce ICAM-1 and VCAM-1 expression. We then tested whether Yoda-1 could mimic the effect of shear stress on doxorubicin-induced cytotoxicity. Both laminar flow and Yoda-1 treatment of static cultures increased the cytotoxicity of doxorubicin. These findings show that Piezo-1 activation with Yoda-1 in static culture leads to an endothelial cell phenotype that mimics endothelial cells under laminar flow. Pharmacological activation of Piezo-1 may be a useful approach to mimic constant shear stress in static cultures, which may improve endothelial drug discovery and toxicity testing.

## Graphical abstract

**Figure:**
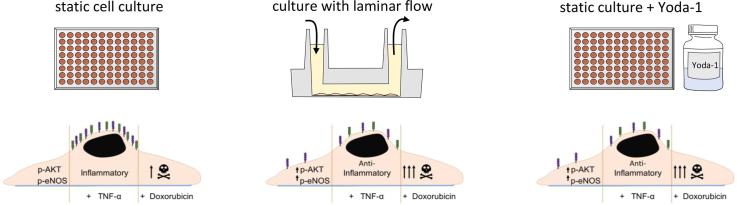

## 1 Introduction

Endothelial cells play a key role in vascular homeostasis, including regulation of vascular tone, inflammation and haemostasis [1]. Endothelial dysfunction is strongly associated with proinflammatory and prothrombotic disease states, such as the initiation and progression of atherosclerosis [2]. Endothelial dysfunction is promoted by risk factors associated with cardiovascular disease, including diabetes, hypercholesterolemia, hypertension and smoking [3]. In addition, the role of endothelial damage in many adverse drug reactions and in cardiovascular toxicity is being increasingly recognised particularly during chemotherapy [4], The endothelium is therefore an attractive therapeutic target and an important drug safety concern.

Endothelial cells in the normal circulation are exposed to haemodynamic forces including shear stress, a frictional force acting parallel to the wall [5]. Although static culture of endothelial cells *in vitro* has yielded invaluable insights into endothelial cell activation and leukocyte recruitment during inflammation [6], [7], shear stress influences the structure and function of endothelial cells and alters gene expression [8]. Constant laminar shear stress has been reported to be anti-inflammatory, for example by reducing the increase in vascular cell adhesion molecule-1 (VCAM-1) expression in response to tumour necrosis factor-alpha (TNF-α) [9], [10], [11].

Including shear stress may improve future endothelial cell drug discovery or toxicity screening and increase clinical trial success rates. A range of methods have been developed to apply shear stress to endothelial cell cultures *in vitro*, including parallel-plate flow chambers, cone-and-plate viscometers, orbital shakers and microfluidics [12], each having advantages and disadvantages. An important drawback with many is the challenge in scaling up for high-throughput screening.

Recently, Piezo-1, a calcium ion channel activated by shear stress, was identified as a pivotal player in vascular mechanotransduction [13], [14]. In many studies, knock-down or partial removal of Piezo-1 resulted in the endothelium failing to align to the direction of flow [15], [16], [17], [18], [19]. Piezo-1 can be selectively activated through the small molecule Yoda-1 in both *in vitro* and *in vivo* settings [20], [21]. Studies with purified Piezo-1 and Piezo-2 protein within lipid bilayers, or Piezo-1 or Piezo-2 heterologously expressed in HEK-293 cells, concluded that Yoda-1 did not activate the highly homologues receptor paralogue Piezo-2 [21], [22]. Currently, no off-target interactions of Yoda-1 has been described. *In vitro*, acute administration of Yoda-1 mimicked the effect of shear stress with activation of nitric oxide synthase in endothelial cells and induced vasodilatation of isolated arteries [19].

In this study, we investigated whether Yoda-1 treatment in static culture could mimic the effect of laminar shear stress on endothelial cell inflammation, and endothelial cytotoxicity in response to the chemotherapy agent, doxorubicin. We propose that pharmacological activation of Piezo-1 may be a useful approach to mimic constant shear stress in static cultures, which may improve endothelial drug discovery and toxicity testing.

## 2 Materials and methods

### 2.1 Cell culture

Human umbilical cord vein endothelial cells (HUVEC) (Promocell, c-12203), derived from pooled donors, were cultured in endothelial cell growth medium (PromoCell, Heidelberg, Germany; c-22010), supplemented with 35 µg/mL gentamycin (Sigma Aldrich, Poole, UK), 2% v/v foetal calf serum (FCS; Sigma Aldrich) and endothelial cell growth supplements (Promocell, c-39215) at 5% CO_2_ and 37 °C. HUVEC were used between passage 4-6. HUVEC were grown on tissue culture-treated plastic-ware.

### 2.2 HUVEC culture under shear stress

HUVEC were seeded into a µ-slide VI^0.4^ (ibidi; Thistle Scientific, Glasgow, UK) at 6x10^4^ cells/channel and left to adhere for 2 h prior to the addition of flow through the connection of a perfusion pump set (ibidi, cat# 10902). The perfusion system is a computer-operated positive air pressure pump (ibidi). HUVEC were exposed to a continuous shear rate of 5 dyn/cm^2^, according to the manufacturer’s instructions. To generate the low shear conditions used for comparison, a non-continuous shear rate of 0.5 dyn/cm^2^ was employed with a cycle lasting approximately 1 min. This was conducted every 2 h to refresh media within the µ-slide VI^0.4^. HUVEC were maintained at 5% CO_2_ and 37 °C while under shear stress.

### 2.3 Flow cytometry

HUVEC monolayers were pre-treated with Yoda-1 (Tocris/Bio-Techne, Bristol, UK) for 24 h. Where indicated, TNF-α (R&D systems / Bio-Techne, Abingdon, UK) (100 U/mL) was introduced at the 20-hr time point. Endothelial cells were detached from cell culture plastic via protease release, trypsin (Sigma Aldrich). Samples were stained with anti-VCAM-1 PE-Cy7 (cat# 25-1069-41, 1:100) and anti-ICAM-1-FITC (cat#BMS108F1, 1:100). Samples were analysed using a BD Accuri C6 flow cytometer. An endothelial positive gate was determined using anti-CD31-APC (cat#17-0319-42, 1:100). All antibodies for flow cytometry were purchased from Thermo Fisher Scientific (UK).

### 2.4 Immunofluorescence

HUVEC were grown to confluence in ibidi µ-slide 8 well coverslips (cat# 80826). In experiments to assess cell death, a cell viability 660 stain (eBioscience, cat# 65-0864-18) was used at 1:1000 for 20 min prior to 4% paraformaldehyde fixation (Alfa Aesar, Thermo Fisher Scientific, Heysham, UK). After washing with phosphate-buffered saline (Sigma Aldrich), samples were incubated overnight with anti-VE cadherin (Cell Signaling Technology, MA, USA, cat# 2500, 1:200). The primary antibody was removed, and fluorophore-conjugated anti-rabbit (Thermo Fisher Scientific, cat# 11-4839-81, 1:300) was applied alongside 2.5 µg/ml bisbenzimide (Sigma Aldrich). Fluorescent images were acquired by confocal scanning laser microscopy (Leica SP5). Images were analysed using the free platform FIJI [23], using the ‘Analyse particles’ function to count nuclei.

### 2.5 Western blotting

Lysates were made in RIPA lysis buffer (0.607 g Tris, 0.876 g NaCl, 0.1 g SDS, 0.5 g sodium deoxycholate, 1 ml Triton X-100 in 49 ml H_2_0). Protein inhibitor cocktail mix (Sigma Aldrich, cat# P2714) was added to prevent protein degradation. Prior to gel loading, lysate concentrations were established via Bradford assay (Sigma). 10 µg of HUVEC lysates were loaded per condition and separated by SDS-PAGE on either an 8 or 12% acrylamide gels under reducing conditions. Proteins were transferred to a PVDF membrane (MerckMillipore). Membranes were blocked with bovine serum albumin (5% w/v; 1 h; room temperature) in Tris-buffered saline containing 0.1% Tween-20 (TBST), after which membranes were incubated with primary antibodies at 4 °C for overnight incubation, anti-p-eNOS (Cell Signaling Technology, cat# 9571, 1:1000) and anti-p-AKT (Cell Signaling Technology, cat# 4060, 1:1000). After washing with TBST and subsequent milk block (5% w/v; 1 h), membranes were incubated for 2 h at room temperature with anti-rabbit HRP, or anti-mouse HRP (both Cell Signaling Technology, 1:5000 and 1:10,000 respectively). When appropriate, anti-beta tubulin (Abcam, Cambridge, UK; cat# Ab7291, 1:2500) was used as loading control. Images were collected through chemiluminescent substrate chemistry (Thermo Fisher Scientific Cat# 34577) and documented using a scanner (Gene Gnome, Syngene).

### 2.6 ATP cell viability assay

HUVEC were seeded at 1.5 × 10^4^ cells/well in a 96-well plate. HUVEC were grown for 12 h prior to treatment with Yoda-1 (or their appropriate vehicle controls). The cell viability assay (Promega, UK; cat# G7572) was conducted according to the manufacturer’s instruction.

### 2.7 Image analysis

The open source image analysis platform FIJI was used. The directionality plugin was used to measure f-actin directionality. HUVEC were manually traced using the free-hand tool to determine the elongation ratio.

### 2.8 Statistical analysis

In each figure panel, each circular symbol corresponds to an independent experiment. The mean ± SD is also shown. Statistical analysis was conducted using GraphPad Prism v5. All statistical tests performed can be found within the relevant figure legends.

## 3 Results

### 3.1 Yoda-1 mimics the effect of laminar shear stress on endothelial cells

**Fig. 1:**
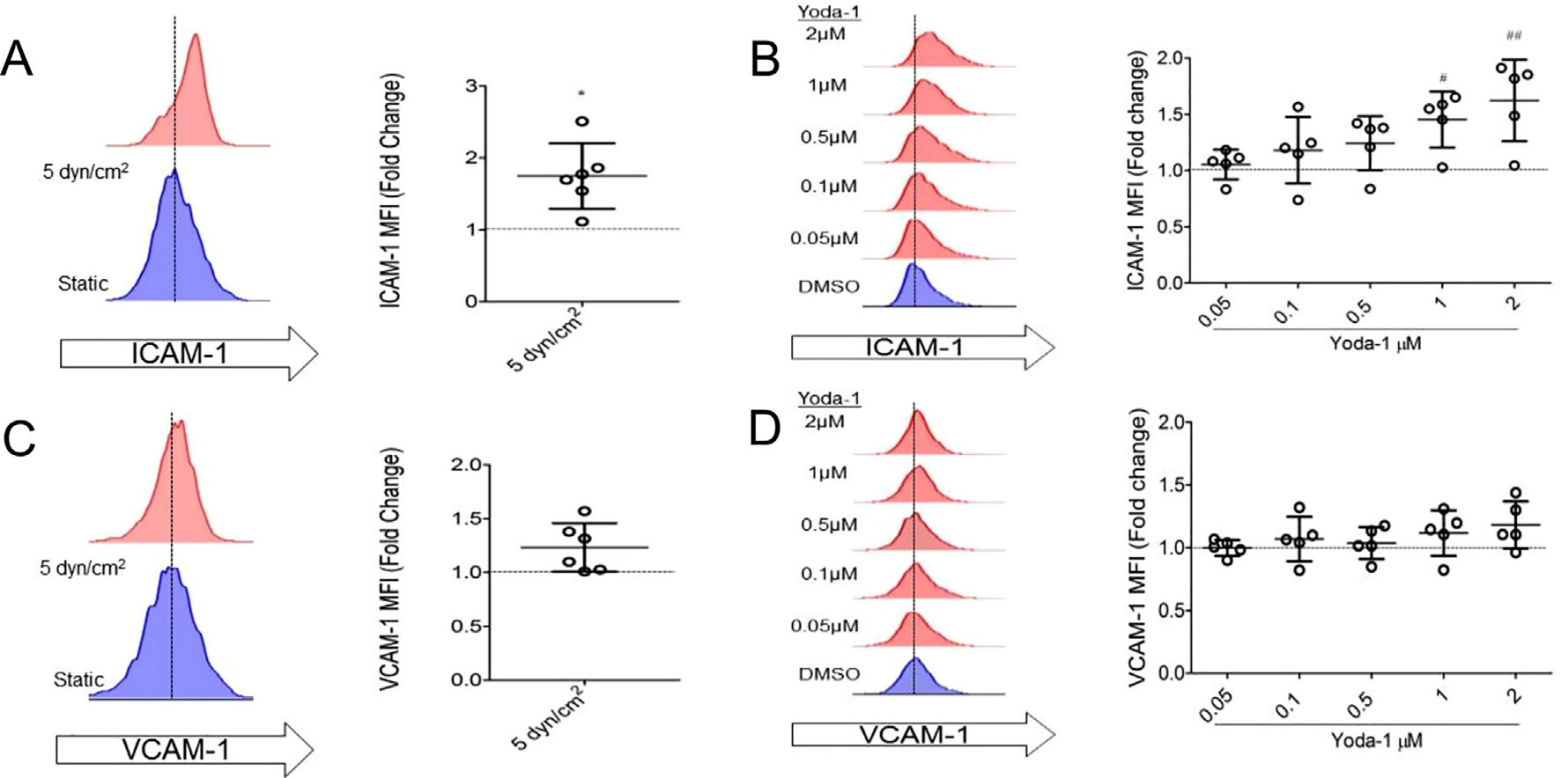
Yoda-1 treated endothelium mimic phenotypes observed in endothelium under laminar shear stress. (A–D) HUVEC were cultured under laminar flow with a constant shear stress of 5 dyn/cm^2^, or under static conditions using a concentration range of Yoda-1, for 24 h. ICAM-1 (A-B) or VCAM-1 (C–D) surface levels were detected by flow cytometry. Representative histograms from a single experiment are shown, followed by median fluorescence intensity (MFI) normalised to their appropriate static culture control. Each circular point represents one independent experimental repeat recorded on a different day. (A, C) Two-tailed non-parametric test conducted. *P < 0.01. (B, D) One-way ANOVA with Dunnett’s post test conducted. ##P < 0.001 ###P < 0.001.

**Fig. 2:**
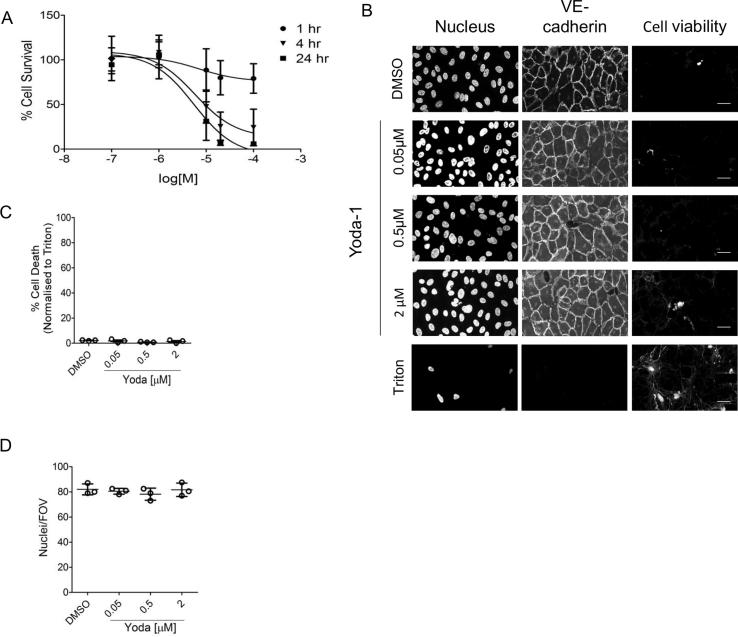
Establishing a working concentration range for Yoda-1. (A) HUVEC were exposed to a concentration range, between 100 µM and 0.1 µM, of Yoda or with the appropriate DMSO vehicle control for 1, 4 or 24 h. Cell death was quantified using an ATP luminescence commercial kit. (B) HUVEC were stimulated with Yoda-1 for 24 h then incubated with a cell viability dye (blue) prior to fixation. To visualise the nuclei and junctions, cells were incubated with bisbenzimide (red) and anti-VE cadherin (green), respectively. Representative confocal images shown. Scale bar = 20 μm. Three independent experiments with three random fields of view (FOV) were quantified per treatment, and % dead cells were normalised against 0.2% (v/v) Triton x 100 treatment. (C–D) Yoda-1-treated HUVEC were imaged and nuclei/FOV counted per treatment. Six random FOV were quantified for each individual repeat. (For interpretation of the references to colour in this figure legend, the reader is referred to the web version of this article.)

The effects of Yoda-1 on HUVEC in static culture for 24 h were compared with the effect of culturing HUVEC under laminar flow conditions for the same time. Culture under constant shear stress (5 dyn/cm^2^) increased surface expression of ICAM-1 by a mean average of 1.77 ± 0.19 fold increase (p < 0.05, n = 5), but not VCAM-1 (Fig. 1 A, C), as has been previously described [24]. Yoda-1 treatment for 24 h mimicked this response, inducing a concentration-dependent increase in ICAM-1 surface expression. 1 μM Yoda-1 significantly increased ICAM-1 levels to 1.45 ± 0.11-fold higher than the DMSO (vehicle) control (Fig. 1 B, D). VCAM-1 surface expression was not affected. Yoda-1 treatment for this time, (and the concentration range used in Fig. 1) had no effect on HUVEC viability, measured by total ATP levels. Although, concentrations higher than 10 μM caused a significant proportion of cell death after 4 h. Parallel cell viability testing through confocal microscopy confirmed that the Yoda-1 up to 2 μM was non-cytotoxic. Incubation of HUVEC with Yoda-1 (0.05–2 μM) showed no effect on cellular proliferation in this concentration range (Fig. 2).

**Fig. 3:**
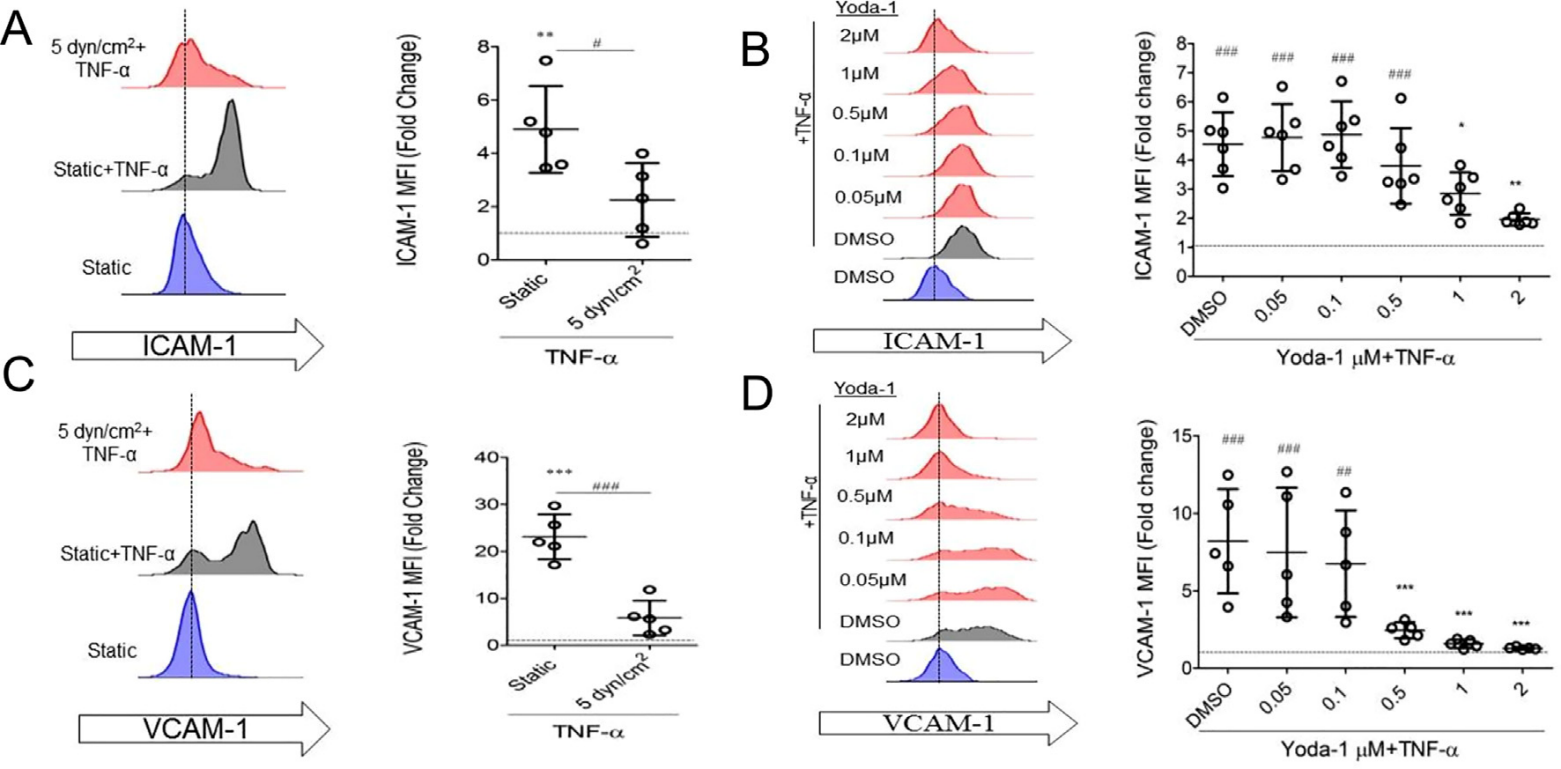
Yoda-1 and shear stress both produce anti-inflammatory phenotypes in endothelium treated with TNF-α. (A–D) Experiments were performed in a similar manner to Fig. 1, but 100 U/mL TNF-α was introduced at the 20 h point for the remaining 4 h. ICAM-1 (A–B) or VCAM-1 (C–D) surface levels quantified (MFI values normalised against their appropriate unstimulated control). (A,C) One-way ANOVA with Tukey’s post-test. *P < 0.01, ##P < 0.001. (B,D) One-way ANOVA with Dunnett’s post test conducted, compared to DMSO (vehicle) control ##P < 0.001 ###P < 0.0001 or TNF-α alone ***P < 0.0001.

The effects of Yoda-1 and laminar shear stress (5 dyn/cm^2^) on the response to the pro-inflammatory cytokine, TNF-α, were also compared. HUVEC were cultured under laminar flow for 20 h, followed by stimulation with TNF-α (100 U/ml) for a further 4 h, also under laminar flow. Alternatively, HUVEC were treated with Yoda-1 for 20 h, followed by stimulation with TNFα for 4 h in the continued presence of Yoda-1. Again, Yoda-1 treatment mimicked the effect of shear stress. Both shear stress and Yoda-1 suppressed the TNF-α-induced surface expression of ICAM-1 and VCAM-1, relative to static controls (Fig. 3A–D). The effect of Yoda-1 was concentration dependent, with an IC_50_ of 1.7 µM and 0.17 µM for ICAM-1 and VCAM-1, respectively. Since 1 µM Yoda gave the closest results to the 5 dyn/cm^2^ flow data, this concentration was chosen for the following experiments.

**Fig. 4:**
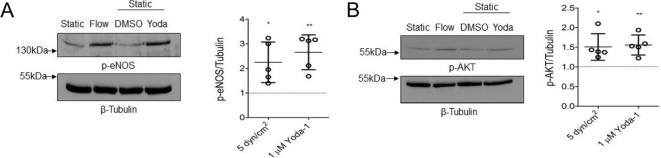
Endothelium treated with either Yoda-1 or shear stress both increase phosphorylation of AKT and eNOS. HUVEC were stimulated for 24 h with either 1 µM Yoda-1 or 5 dyn/cm^2^. Lysates were subjected to immunoblotting with anti-phospho eNOS (Ser 1177) (A) or anti-phospho Akt (Ser 473) (B). Anti-β Tubulin was used as a loading control. Representative blots and quantification by densitometric analysis are shown. β-Tubulin-corrected data are normalised against their appropriate static control lysate (dotted line). One-way ANOVA with Tukey’s post-test. *P < 0.01, **P < 0.001.

We also confirmed that Yoda-1 at this concentration also mimicked the effect of laminar shear stress on phosphorylation of endothelial nitric oxide synthase (eNOS) and its upstream kinase, Akt (Fig. 4). There was no significant difference between the phosphorylation levels of either eNOS and Akt in lysates from laminar sheared (5 dyn/cm^2^) or statically cultured 1 µM Yoda-1-treated HUVEC. This has been previously described following acute treatment with Yoda-1 (under 30 min), but not for more extended treatment as used here [19].

**Fig. 5:**
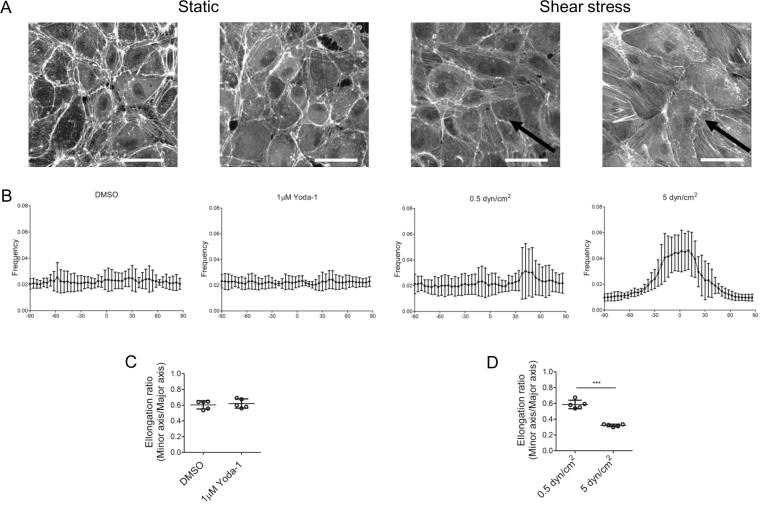
Yoda-1 treatment does not induce f-actin alignment and cell elongation. HUVEC were treated under static conditions for 24 h (1 μM Yoda-1 or DMSO), or under shear stress continuously (5 dyn/cm^2^) or a non-continuous low shear stress (0.5 dyn/cm^2^; 1 min flow cycle per 2 h). To visualise f-actin, cells were stained with phalloidin. (A) Representative confocal pictures for each condition. Arrow indicates the direction of media flow across the endothelium. Scale bar = 25 μm. (B) Directionality of f-actin fibres (relative to the direction of media flow when indicated by a black arrow) For each condition, three randomly selected fields of view were chosen for analysis. Data represent five independent repeats. (C–D) Elongation ratio (cell minor axis length)/ (cell major axis length). Non-paired students *t*-test conducted for the respective static and shear stress conditions ***P < 0.0001.

Together this works shows that Yoda-1 treatment can mimic the effect of continuous laminar shear stress on endothelial cells. One response characteristic of shear stress is that f-actin fibres align parallel to the direction of flow [25]. This was not observed when HUVEC in static culture were treated with 1 μM Yoda-1 (Fig. 5). This is not surprising, since there was no direction of flow towards which the cells could align. Cell bodies were not elongated under Yoda-1 treatment. In contrast, continuous shear stress of 5 dyn/cm^2^ dramatically altered both cell body elongation and f-actin alignment (Fig. 5).

### 3.2 Yoda-1 mimics the effect of laminar shear stress of endothelial cytotoxicity

**Fig. 6:**
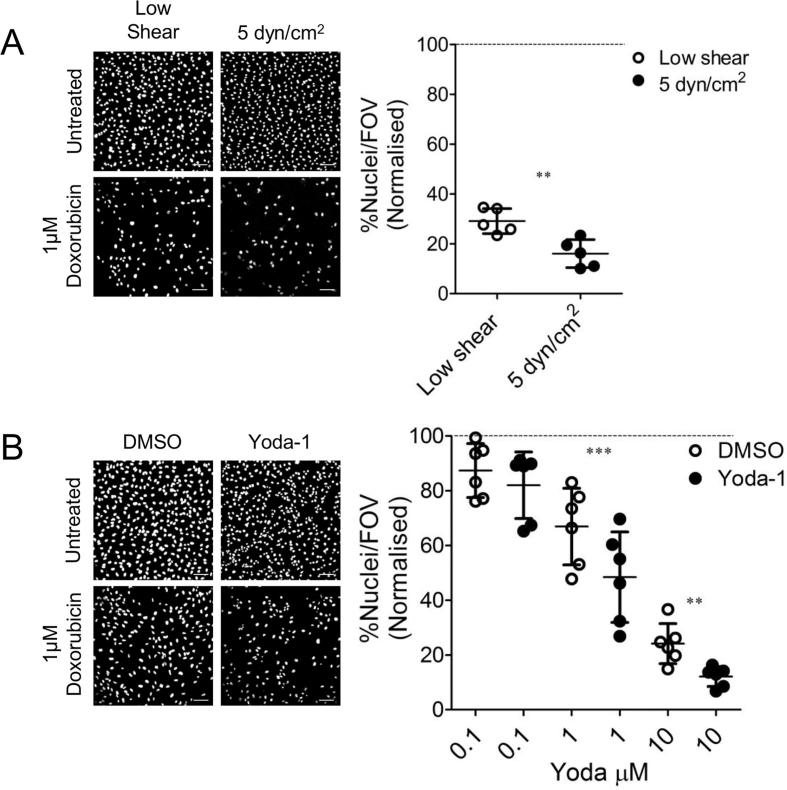
Yoda-1 and laminar shear stress increase endothelial cytotoxicity in response to doxorubicin. (A) HUVEC were either placed under 5 dyn/cm^2^ or low flow conditions (see materials and methods for description) for 24 h prior to the addition of 1 µM doxorubicin. Constant flow then continued for a further 24 h. Representative confocal images of stained nuclei are shown (Scale bar = 100 μm). Per condition 6 random fields of view were selected and imaged through confocal microscopy. Data represent the number of nuclei present in the field of view (FOV) as a percentage of the appropriate no doxorubicin control. Each circular point represents one independent experimental repeat recorded on a different day. Dashed line indicates normalisation. Two-tailed non-paired *t*-test conducted, **P < 0.001. (B) HUVEC were grown either with 1 μM Yoda-1 or the DMSO (vehicle) control for 24 h, then challenged with doxorubicin for a further 24 h. Yoda-1 or DMSO was not removed during this period. HUVEC were then imaged as described above. Scale bar = 100 μm. Two tailed paired *t*-test conducted, **P < 0.001, ***P < 0.0001.

To test whether Yoda-1 treatment could mimic the effect of laminar shear stress on drug toxicity, we investigated the effect of the chemotherapy agent doxorubicin. Doxorubicin reduced the viability of HUVEC cultured under static conditions in a concentration-dependent manner (Fig. 6). HUVEC were cultured under shear stress for 24 h followed by treatment with the chemotherapy agent (or vehicle control) for a further 24 h with continuing shear stress. For comparison, HUVEC were also cultured in the channel slides under very low shear conditions (0.5 dyn/cm^2^ for 1 min every two hours, to allow exchange of media). Interestingly, the cytotoxicity of doxorubicin was increased by 5 dyn/cm^2^ shear stress compared to under low flow conditions (Fig. 6A), with only 16.0 ± 2.5% of nuclei remaining compared to 29.1 ± 2.2% (n = 5, p < 0.05). The same was observed in static culture when Yoda-1 was used in comparison to the vehicle alone. HUVEC were treated with Yoda-1 for 24 h followed by treatment with the chemotherapy agent for a further 24 h (with continuing presence of Yoda-1). Again, the cytotoxicity of doxorubicin was increased (Fig. 6B) in the presence of Yoda-1 compared to DMSO. These data therefore show that Yoda-1 treatment can mimic the effect of constant shear stress on the cytotoxicity of doxorubicin.

## 4 Discussion

The endothelium is both a therapeutic target and a potential site of adverse drug reactions [26], [27]. *In vivo_,_* endothelial cells are exposed to shear stress that changes their structure and function [28]. Mimicking *in vivo* conditions such as shear stress on endothelial cells *in vitro* is an important consideration in endothelial drug discovery and toxicity testing, as a means of generating endothelial cells that more closely resemble the *in vivo* endothelium. Since Piezo-1, a mechanotransducer, is required for full endothelial responses to shear stress [15], [16], [17], we hypothesised that activation of Piezo-1 by a small molecule would mimic the effect of shear stress of endothelial responses to inflammation and drug treatment. The primary goal of this work was to test this hypothesis.

To do this, we used Yoda-1, previously-characterised small molecule activator of Piezo-1. Syeda and colleagues demonstrated that Yoda-1 can activate Piezo-1 in artificial lipid bilayers without other accessory proteins. Importantly, Yoda-1 did not activate Piezo-2, a homologous protein which is also expressed in endothelial cells [29], [21]. *In vitro*, activation of nitric oxide synthase by shear stress on endothelial cells could be mimicked by acute administration of Yoda-1 [19]. Therefore Yoda-1, which is active *in vitro* and with no currently identified off-target interactions was chosen as approach to activate Piezo-1. However, a concern was that although Yoda-1 has been used to acutely mimic shear stress (over tens of minutes; [16], [19]), it was not clear whether cultured endothelial cells would tolerate this over longer culture (24–48 h). The concentrations of Yoda-1 used (up to 2 µM) did not cause cytotoxicity or alter HUVEC proliferation, whereas higher concentrations (10–100 µM) caused substantial cytotoxicity and compromise of the HUVEC monolayer.

First, we tested whether Yoda-1 could mimic the well-reported effects of shear stress of expression of the endothelial adhesion molecules, ICAM-1 and VCAM-1. Two effects of shear stress have been previously reported: an increase in ICAM-1 expression in unstimulated endothelial cells, and inhibition of the ability of TNF-α to increase ICAM-1 and VCAM-1 expression. We chose to compare Yoda-1 (1 μM) to a shear stress of 5 dyn/cm^2^ as this is physiologically compatible for HUVECs [30].

Culture under shear stress leads to an increase in ICAM-1 mRNA and cell-surface expression. This has been observed across a wide range of shear stress (1–15 dyn/cm^2^) [24], [31]. Although the physiological relevance of this relatively small increase in basal ICAM-1 is unclear, it does appear to be selective as other cell adhesion molecules, notably VCAM-1, are not affected. Our work mirrors this basal increase in cell surface levels of ICAM-1 when the endothelium was cultured under 5 dyn/cm^2^ shear stress. Importantly, ICAM-1 was also increased to a similar extent in a concentration-dependent manner when exposed to Yoda-1 in static culture. Neither 5 dyn/cm^2^ shear stress nor Yoda-1 increased VCAM-1 expression. This indicates that Yoda-1 can modulate basal ICAM-1 expression in a similar manner seen in our shear stress conditions and in previous reports. Modulation of basal ICAM-1 is not only a marker of the effect of shear stress, it may also be directly relevant to the action of some therapeutics. For example, delivery of drugs to the endothelium can be achieved by using nanocarriers targeted to ICAM-1 [32], [33]. This can also be affected by other phenotypical responses of endothelial cells to flow, such as reorganisation of action stress fibres and modulation of the glycocalyx [34], [35], further emphasising the importance of considering the effect of shear stress of endothelial cells during drug development and toxicity screening.

In addition in upregulation of ICAM-1, Yoda-1 (1 μM) induced phosphorylation of eNOS and its upstream kinase, Akt, in a very similar manner to culture under shear stress for the same period. These responses are not restricted to acute treatment but are maintained over 24 h. Together, these data show that Yoda-1 (1 μM) induced the same responses in endothelial cells as does shear stress.

Under inflammatory conditions, laminar shear stress is considered anti-inflammatory. Laminar shear stress markedly reduces the upregulation of ICAM-1 and VCAM-1 in response to inflammatory cytokines such as TNF-α, reducing leukocyte capture [10], [11]. Consistent with this, culture under constant shear stress in our system decreased the TNF-α-induced expression of VCAM-1 and ICAM-1. This could also be mimicked by Yoda-1. Culture with Yoda-1 reduced the TNF-α-induced upregulation of ICAM-1 and VCAM-1 in a concentration-dependent manner and to a similar extent to 5 dyn/cm^2^ shear stress. Together, these data show that Yoda-1-treated endothelial cells faithfully show the same anti-inflammatory phenotype as cells cultured under shear stress.

Having demonstrated that Yoda-1 effectively mimics the effect of shear stress on the expression of cell adhesion molecules, we next examined the implications for testing drug cytotoxicity. Doxorubicin is a cytotoxic chemotherapeutic agent through inhibition of topoisomerase II and disruption of DNA replication and transcription, ultimately leading to cell death [36]. Doxorubicin causes death of endothelial cells, leading to vascular toxicity, this may also contribute to its cardiotoxicity [37]. Although laminar shear stress has been widely reported to have a protective effect from numerous damaging stimuli [38], [39], it increases endothelial cytotoxicity in response to doxorubicin [40]. This may be through an increase in reactive oxygen species leading to activation of p53 and the pro-apoptotic protein BAX. We therefore used doxorubicin as a model of how drug toxicity may be underestimated if the effect of shear stress is not considered *in vitro*.

Doxorubicin cytotoxicity was enhanced by Yoda-1 in a concentration-dependent manner and was most clearly seen at the same concentration that best mimicked the effect of 5 dyn/cm^2^ shear stress on expression of ICAM-1 and VCAM-1. Doxorubicin cytotoxicity was also enhanced by culturing HUVEC under 5 dyn/cm^2^ shear stress, consistent with previous studies. The extent of cell loss in the two experimental conditions are difficult to compare directly, with a much greater degree of cell loss in flow chamber slides than in static culture wells. This may reflect washing away of dying or stressed endothelial cells in the chamber slides, where 5 dyn/cm^2^ shear stress was compared to ‘low shear’ of intermittent 0.5 dyn/cm^2^ for 1 min every 2 h to allow exchange of the very small volume of culture medium in these channels. After 24 h HUVEC alignment with the flow direction was observed at 5 dyn/cm^2^ but not with non-continuous shear stress of 0.5 dyn/cm^2^ (Fig. 5). In preliminary experiments, we found that this low shear exchange of culture medium was necessary to prevent extensive death of HUVEC without doxorubicin over 48 h, though it does mean that the two experimental conditions (Yoda-1 and flow) are not directly comparable. Nonetheless, the Yoda-1 mimics the effect of shear stress in that doxorubicin cytotoxicity is increased compared to static culture alone.

Our approach aims to mimic the effect of shear stress on endothelial cells and so to improve their physiological relevance. However, there are many differences between standard static culture and the physiological situation *in vivo*. Laminar flow is just one of these, albeit a very important one. Blood cells, other vessel wall components (e.g. smooth muscle cells, pericytes, different extracellular matrix components), and other physical factors such as stretch, will all play an important role in the response of the endothelium. The contribution of each of these could, in principle, be added to this model. However, it will be important to continue to strike an appropriate balance between detailed modelling of the *in vivo* situation, and producing a simple and robust model that is suitable for high-throughput screening in endothelial drug discovery and toxicity testing.

Our study was also confined to HUVEC, with the aim to model small veins and post capillary venules, as these vessel locations are responsible for a large degree of leukocyte binding through ICAM-1 and VCAM-1 [41]. It was also appropriate since HUVEC are a widely used and well-characterised ‘model’ endothelial cell. However, it is possible that endothelial cells derived from alternative vascular beds could respond to Yoda-1 differently, differential expression of Piezo-1 has not been explored. Further work should explore whether Yoda-1 (in a concentration dependent manner) can accurately mirror phenotypes observed in endothelial cells naturally exposed to higher (>5 dyn/cm^2^) and lower (<5 dyn/cm^2^) shear rates. Indeed, it would be appropriate for screening projects using endothelial cells of other origins to first examine a range of concentrations of Yoda-1 and compare against laminar flow experiments conducted at the most appropriate shear stress.

In summary, we have demonstrated that the small molecule activator of Piezo-1, Yoda-1, can be used to mimic shear stress in endothelial cells in static culture We have shown that Yoda-1 mimics the effect of venous shear stress on expression of inflammatory cell adhesion molecules, ICAM-1 and VCAM-1, and on cytotoxicity to the vascular-damaging chemotherapy agent, doxorubicin. Pre-treatment of static endothelial cell cultures with Yoda-1 is much more amenable to high-throughput screening that microfluidic approaches, providing a rapid and simple way to improve endothelial drug screening. Although we would still recommend further analysis of drug action with *in vitro* microphysiological systems or *in vivo* models; our approach might allow these more laborious approaches to be focused on specific drugs of interest. One limitation of our approach is that although Yoda-1 appears to effectively mimic constant shear stress from laminar flow, the endothelial response to turbulent flow is often very different and pathologically-relevant [42]. As our understanding of endothelial mechanotransduction increases, further additional small molecule activators of other mechanosensitive proteins, such as some TRP channels and GPCRs [43], may be useful for mimicking different flow patterns. Until then, this study demonstrates the concept that small molecules can be used to induce more physiological responses in static endothelial cell culture by activating mechanosensitive pathways.

## Author contributions

J. Davies and M. Harper designed the study. All authors performed experiments and analysed data. The manuscript was written by J. Davies and M. Harper. The paper was reviewed by all authors.
